# Molecular Characterization of Herpesviral Encephalitis in Cetaceans: Correlation with Histopathological and Immunohistochemical Findings

**DOI:** 10.3390/ani12091149

**Published:** 2022-04-29

**Authors:** Eva Sierra, Antonio Fernández, Carolina Fernández-Maldonado, Simona Sacchini, Idaira Felipe-Jiménez, Simone Segura-Göthlin, Ana Colom-Rivero, Nakita Câmara, Raquel Puig-Lozano, Anna Maria Rambaldi, Cristian Suárez-Santana, Manuel Arbelo

**Affiliations:** 1 Atlantic Cetacean Research Center, Veterinary Histology and Pathology, Institute of Animal Health (IUSA), Veterinary School, University of Las Palmas de Gran Canaria (ULPGC), Trasmontaña, s/n, 35413 Arucas, Las Palmas, Spain; eva.sierra@ulpgc.es (E.S.); Spain; carogue38@hotmail.com (C.F.-M.); simona.sacchini@ulpgc.es (S.S.); idaira.felipe101@alu.ulpgc.es (I.F.-J.); siimone.andrea@gmail.com (S.S.-G.); ana.colom101@alu.ulpgc.es (A.C.-R.); nakita.camara101@alu.ulpgc.es (N.C.); raquelpuiglozano@gmail.com (R.P.-L.); annamaria.rambaldi@gmail.com (A.M.R.); cristian.suarez@ulpgc.es (C.S.-S.); manuel.arbelo@ulpgc.es (M.A.); 2 Seashore Environment and Fauna, C/Sevilla 4, 11380 Tarifa, Cádiz, Spain

**Keywords:** alphaherpesvirus, gammaherpesvirus, encephalitis, meningitis, malacia, haemorrhages, intranuclear inclusion bodies, morbillivirus, superinfection, qPCR

## Abstract

**Simple Summary:**

In this study we describe the molecular and pathological characteristics of alpha- and gamma-herpesvirus infection of the central nervous system of stranded cetaceans and correlate them with viral load, immunohistochemical findings and biological data such as age, sex, and the presence of co-infections. The viruses (alpha- and gamma-herpesvirus) were detected in twelve out of 103 analysed stranded cetaceans and were associated with a wide range of histopathological lesions, as previously described for these and other species. In five out the twelve animals, lesions were severe enough (malacia, neuronal necrosis and neuronophagia) to cause death. Intranuclear inclusions bodies were present in brain tissue samples from half of the HV-positive animals, indicating that the injury was due to an infective agent belonging to a group of filterable viruses. These results are in accordance with immunohistochemical findings, as all the brain tissue samples with INIBs were immunolabeled with Anti-HSV1. Males, juveniles, and calves were predominantly infected among the analysed cetaceans and a 41.6% (5/12) incidence of co-infections in the brain was detected, with three animals co-infected with Dolphin Morbillivirus (DMV). In this study, we present, to the best of our knowledge, the first histopathological evidence of superinfection between HV and DMV pathogens in brain tissue.

**Abstract:**

Herpesviruses are causative agents of meningitis and encephalitis in cetaceans, which are among the main leading known natural causes of death in these species. Brain samples from 103 stranded cetaceans were retrospectively screened for the presence of herpesvirus DNA in the brain. Molecular detection of Cetacean Morbillivirus was performed in HV positive brain cases. Histopathologic evaluation of brain samples included the presence or absence of the following findings (*n* = 7): meningitis, perivascular cuffings, microgliosis, intranuclear inclusion bodies, malacia, neuronal necrosis and neurophagic nodules, and haemorrhages. Histological evidence of the involvement of other etiological agents led to complementary analysis. We detected the presence of alpha and gamma-HVs in 12 out of 103 (11.6%) brain samples from stranded cetaceans of five different species: one bottlenose dolphin, six striped dolphins, three Atlantic spotted dolphins, one Cuvier’s beaked whale, and one common dolphin. Pathogenic factors such as viral strain, age, sex, and the presence of co-infections were analysed and correlated with the brain histopathological findings in each case. Herpesvirus was more prevalent in males, juveniles, and calves and a 41.6% incidence of co-infections in the brain was detected in our study: three with Dolphin Morbillivirus, one with *Staphilococcus aureus septicaemia* and one with *Brucella* spp.

## 1. Introduction

Herpesviruses (HVs) are generally host-specific widespread pathogens responsible for a wide range of diseases, from unapparent infections to mild, moderate or severe disease, depending on the inherent characteristics of the host and biological characteristics of the virus [1]. All HVs share common biologic properties, such as common morphology and the ability to establish persistent infections, although some differences are related to host specificity, tissue tropism, replication kinetics and pathogenic potential [2]. Indeed, HVs can be highly pathogenic in a variety of conditions, such as a compromised immune system or aberrant/naïve host [3], environmental circumstances [4], co-infection [1], and/or different pathogenic viral strains [5].

HVs [*Alpha* (α)- and *Gamma* (γ)-*herpesvirinae* subfamilies] have been detected in eight families in the suborder Odontoceti (toothed whales): Delphinidae, Iniidae, Kogiidae, Monontidae, Phocoenidae, Physeteridae, Pontoporiidae and Ziphiidae [6,7,8,9,10,11,12,13,14]; and in the Balaenoptera genus (suborder Mysticeti) [15]. α-HVs have been commonly associated with skin lesions and systemic infections [8,9,16,17,18], whereas γ-HVs have been frequently found in genital lesions [7,11,13,19,20,21,22,23,24]. In addition, α- and γ-HV infection of the central nervous system (CNS) has been described in a few cetacean species. More specifically, the characteristic pathological changes of alphaherpesviral encephalitis have been reported in harbour porpoises (*Phocoena phocoena*) [11,25], in a common bottlenose dolphin (*Tursiops truncatus*) [26], in a common dolphin (*Delphinus delphis*) [27], in striped dolphins (*Stenella coeruleoalba*) [17,27], and in Atlantic spotted dolphins (*Stenella frontalis*) [27]. However, several cases of α-HV infection in the CNS with histopathological changes not directly attributable to these viruses or in the absence of lesions have also been described [11,17,27,28]. Classical CNS lesions associated, but not exclusively, with the presence of α-HVs consist of meningeal mononuclear cell infiltrates, lymphoplasmacytic perivascular cuffs, microgliosis, intranuclear inclusion bodies (INIBs), and neuronal necrosis and/or associated focal neuronophagic nodules [11,25,26]. Some of these lesions (specifically lymphoplasmacytic perivascular cuffs) have been recently described associated with γ-HV detection in the brain of striped and common bottlenose dolphins [29,30].

Several studies have reported different frequencies in HVs infection in stranded cetaceans. In systematic surveys, HV has been detected in 80.85% (38/47) of cetaceans from the Mediterranean (Valencia, Spain) [31], in 78.57% (11/14) of striped dolphins from Cantabria (Spain) [30], in 14.5% of beaked whales from the Canary Islands (Spain) [32], in 7.8% (14/179) of cetaceans from the Portuguese coastline [19], in 5% (4/79) of cetaceans from Japan [12] and in 3.7% (4/109) of cetaceans from Brazil [13]. A percentage of 6.7% (5/74) of HV infection was found in harbour porpoises from the Netherlands when only the CNS was analysed [11].

To better characterize different HV strains associated with encephalitis in cetaceans, we analysed the presence of these viruses in the brain of stranded cetaceans and correlated the pathological descriptions of the brain lesions with the biological data, the detected viral strains, the virus load and the immunohistochemical staining in each case. All the cases included in the present study were diagnosed during routine pathological and cause of death analyses in stranded cetaceans; the analyses were performed at the Division of Histology and Animal Pathology of the Institute for Animal Health (IUSA), Veterinary School, Universidad de Las Palmas de Gran Canaria. Since 1999, prospective studies on all stranded cetaceans in the Canarian Archipelago, and occasionally other geographic regions, have been systematically carried out. In addition, a retrospective study is currently being performed to detect pathogens potentially associated with brain lesions in banked tissues of stranded cetaceans from the archives of the IUSA.

## 2. Materials and Methods

### 2.1. Evidence of Ethical Approval

Permission for the management of stranded cetaceans was issued by the environmental department of the Canary Islands Government and the Spanish Ministry of Environment. Moreover, neither animal was sacrificed and no experiments were performed with live animals, so ethical review and approval were waived.

### 2.2. Animals, Necropsies, and Tissue Sampling

From 1996 and 2018, 664 cetaceans were found stranded and subjected to necropsies and complete pathological studies in the Canary Islands, Spain. As a part of the screening process for common pathogens in cetaceans, brain samples (*n* = 101; 15.2%) were retrospectively screened for the presence of herpesvirus DNA. Additionally, samples from two stranded cetaceans from Andalusia, Spain, were also screened (104 stranded cetaceans were subjected to complete necropsies and pathological studies in this geographical region from 2011 to 2014) (Supplementary Table S1). The animals identified included eight short-beaked common dolphins (*Delphinus delphis*), ten short-finned pilot whales (*Globicephala macrorhynchus*), ten Risso’s dolphins (*Grampus griseus*), two pygmy sperm whales (*Kogia breviceps*), one Blainville’s beaked whale (*Mesoplodon densirostris*), seven Gervais’ beaked whales (*Mesoplodon europaeus*), one sperm whale (*Physeter macrocephalus*), 27 striped dolphins, eight Atlantic spotted dolphins (*Stenella frontalis*), two rough-toothed dolphins (*Steno bredanensis*), 20 common bottlenose dolphins, and seven Cuvier’s beaked whales (*Ziphius cavirostris*). Fifty animals were males (50/103; 48.5%) and 52 females (52/103; 50.5%), while in one animal the sex could not be determined (1/103; 1%). Ten animals were categorized as calves (10/103; 9.7%), 39 as juveniles (39/103; 37.9%), 52 as adults (52/103, 50.5%), while in two animals the age remained undetermined (1/103; 1.9%).

All the animals were examined and necropsied according to standard procedures [33,34,35,36]. During necropsy, representative samples of skin, longissimus dorsi and rectus abdominis muscles, peritoneum, diaphragm, central nervous system, hypophysis, thyroid gland, adrenal glands, eye, pterygoid sac, tympanoperiotic complexes, tongue, oral mucosa, pharyngeal and laryngeal tonsils, oesophagus, stomach, small and large intestine, anus, liver, pancreas, trachea, lung, heart, aorta, rete mirabile, kidney, ureter, urinary bladder, urethra, lymph nodes, spleen, testicle, penis, prepuce, ovary, uterus, vagina, vulva and mammary gland, were collected and fixed in 10% neutral buffered formalin. The fixed tissue samples were trimmed, routinely processed, embedded in paraffin, sectioned at a 5 µm, and stained with haematoxylin and eosin (HE) for examination by light microscopy.

Fresh unfixed samples (mainly skin, muscle, lung, prescapular, pulmonary, mediastinal and mesenteric lymph nodes, liver, intestine, kidney, spleen, brain) were stored frozen at −80 °C until processed for molecular virology testing. In addition, selective samples were submitted for bacteriological analysis including routine culture and surface plating on routine media and preliminary identification of isolates via the analytical profile index (API) system.

### 2.3. Histopathological Study

Cetacean brains were removed and usually dissected within 24–48 h after necropsy. A superficial sampling of fresh unfixed brain was usually carried out to complement histopathological evaluations with microbiological, virological, and toxicological analyses. The following samples were taken: the cerebral cortex (rostral and caudal), the pons, the cerebellum, and the medulla oblongata. In addition, a cut was made in each hemisphere to expose the lateral ventricles for a biopsy punch inserted in the lateral ventricles to take samples of the caudate nucleus and the thalamus. A sample of the choroid plexus was also taken [37].

Brain cortex lesions were systematically recorded, since they were consistently represented in the sample set. Histopathologic evaluation of brain samples included the presence or absence of the following (*n* = 7): meningitis, perivascular cuffing, microgliosis, INIB, malacia, neuronal necrosis and neuronophagic nodules, and haemorrhages [27]. Lesions affecting other regions and the presence of CNS-associated lesions were also described when present. Necropsy reports and histopathological diagnostic reports of CNS, as well as epidemiologic and biologic data, photographic material, and ancillary diagnostic techniques, were retrieved and further analysed. Histological evidence of the involvement of other etiological agents led to complementary analyses (PCR and/or microbiology).

### 2.4. Molecular Virological Analyses

Approximately 0.5 g of fresh frozen tissue (446 tissue samples), including brain cortex (frontal lobe) (103/103; 100%), lung (85/103; 82.5%), liver (63/103; 61.2%), kidney (63/103; 61.2%), skeletal muscle (58/103; 56.3%), skin (54/103; 52.4%), spleen (51/103; 49.5%), mesenteric lymph node (37/103; 35.9%), intestine (14/103; 13.6%), prescapular lymph node (12/103; 11.6%), pulmonary lymph node (6/103; 5.8%), blood (2/103; 1.9%), thymus (2/103; 1.9%), laryngeal tonsil (2/103; 1.9%), oesophagus (1/103; 0.97%), thyroid gland (1/103; 0.97%), urinary bladder (1/103; 0.97%), oral mucosa (1/103; 0.97%), and reproductive system (1/103; 0.97%) were individually mechanically macerated in lysis buffer and subsequently centrifuged. The DNA/RNA extraction was carried out from each 300 µL macerated sample by the pressure filtration method, using a QuickGene^®^ Mini 80 nucleid acid isolation instrument, using DNA Tissue Kit S (QuickGene, Kurabo, Osaka, Japan) according to the manufacturer’s instructions, with modifications: RNA carrier (Applied Biosystems^TM^, Thermo Fisher Scientific, Waltham, MA, USA) was added during the lysis step [38]. Herpesvirus DNA was detected by conventional nested PCR using degenerate primers designed to amplify a region of the DNA polymerase gene [39]. The PCR products from positive cases were purified using a Real Clean spin kit (REAL) and sequenced (Sanger method). To quantify the viral load of herpesviral-positive samples, a quantitative modified version of the semi-nested conventional PCR was developed in our laboratory. To quantitate herpesviral DNA, a standard curve was obtained from seven consecutive dilutions (dilution factor 1: 10) containing from 10 6 to 10 1 copies/reaction of a commercial positive control (Amplirun Herpes Simplex 1 DNA Control; Vircell, Granada, Spain) with 11000 copies/mL. The amounts of herpesviral DNA in samples were obtained by plotting Ct (cycle threshold) values onto the standard curve.

A BLAST search (www.ncbi.nlm.nih.gov/blast/Blast.cgi (accessed on 27 December 2021)) was conducted to compare the sequenced products with similar DNA polymerase sequences available from other cetacean HVs described in GenBank. A best fit model was selected by the use of the corrected Akaike information criterion (AICc) and Bayesian information criterion (BIC) for nucleotide substitution for a maximum likelihood (ML) analysis using Mega X [40]. A phylogenetic tree was constructed using the ML statistical method with the Tamura 3-parameter model (T92 + G) for nucleotides [40]. A bootstrap resampling (500 replicates) was used to assess the reliability of the tree.

To evaluate the impact of co-infections in herpesviral encephalitis, molecular detection of *cetacean morbillivirus* (CeMV) and Flaviviruses, including the two main lineages of West Nile Virus (WNV), were performed in brain tissue samples of HV-molecularly positive cases from our study. CeMV was diagnosed by four different PCR methods: (a) a reverse transcription (RT) PCR using nested primers targeted at the phosphoprotein (P) gene [17], (b) a one-step RT PCR (RT-qPCR), amplifying a 426-bp conserved region of the phosphoprotein (P) gene [41], (c) a one-step RT-quantitative (q)PCR to detect sequences in a conserved region (192 bp) of the fusion protein (F) gene [38], and (d) a PAN RT-qPCR method based on SYBRN^®^ Green dye that successfully detects GDMV, PWMV and DMV strains and amplifies a 205 bp partial region of the P gene [42]. Several PCR-based diagnostic methods were performed for WNV-L1 [43] and WNV-L2 [44] detection. A quantitative duplex-PCR amplifying a 150 bp fragment of the IS711 gene for the detection of *Brucella* at the genus level and the identification of genotype ST27 was used for the *Brucella* PCR assay [45]. Negative controls (non-template) and amplification-positive controls were added in each PCR protocols described above.

### 2.5. Immunohistochemical Analyses

Immunohistochemical demonstration of the herpesvirus antigen was performed using the Avidin-biotin complex (ABC) method: Formalin-Fixed Paraffin-Embedded (FFPE) tissue sections were mounted on slides pre-coated with Vectabond (Vector), deparaffinized and hydrated in decreasing alcohol baths. Antigen retrieval was carried out with heat-induced epitope retrieval (citrate buffer for 10 min. at 95 °C). Primary antibody (polyclonal Anti-HSV1 antibody (ab9533) provided by Abcam, Cambridge, UK) was diluted 1/75 and incubated in a humid chamber at 4 °C for 18–20 h, followed by blocking of endogenous peroxidase in 0.3% hydrogen peroxide in distilled water for 30 min. Binding between tissue antigens and antibodies was visualized by adding the chromogen 3-Amino-9-Ethylcarbazole (AEC) for 10 min. The presence of a cross-reactive antigen in cetaceans’ brain tissue with an antiserum to human herpesvirus-1 (HSV1) has previously been demonstrated [25]. Positive control included kidney from an HV-positive Blainville’s beaked whale [9].

Immunohistochemical demonstration of the canine distemper virus antigen was performed using the ABC method: FFPE tissue sections were mounted on slides pre-coated with Vectabond (Vector), deparaffinized and hydrated in decreasing alcohol baths and followed by blocking of endogenous peroxidase with distilled water and hydrogen peroxide (6%) for 30 min. Antigen retrieval was carried out with heat-induced epitope retrieval (5 min. at 118 °C in autoclave). Primary antibody (anti-CDV-NP MoAb (provided by VMRD, Pullman, WA, USA)) was diluted 1/100 and incubated in a humid chamber at 4 °C for 18–20 h. Binding between tissue antigens and antibodies was visualized by adding the chromogen 3-Amino-9-Ethylcarbazole (AEC) for 5 min [46]. Positive control included laryngeal tonsil from a CeMV-positive striped dolphin stranded in the Canary Islands in 2019. For negative controls, in both immunohistochemical analyses, sequential sections of the positive control tissues were incubated with nonimmune homologous serums instead of primary antibodies.

## 3. Results

### 3.1. Molecular Results

Molecular methods detected the presence of herpesvirus DNA in the brain of 12 out of 103 (11.6%) animals [17,26,27] and in peripheral organs in additional 13 animals (25 positive animals in total: 25/103; 24.3%) previously reported [17,26,27,32,47] or unpublished. Forty tissue samples (twelve from the brain and 28 from peripheral organs) were molecularly positive to HV. Data (laboratorial reference, species, sex, age, stranding data, stranding location and decomposition code) from brain HV-positive animals are compiled in Table 1 and included data from six striped dolphins (five from the Canary Islands and one from Andalusia), three Atlantic spotted dolphins (all from the Canary Islands), one bottlenose dolphin (from the Canary Islands), one common dolphin (from the Canary Islands), and one Cuvier’s beaked whale (from Andalusia). When considering the percentage of HV-positive cases in each cetacean species, we found the highest positive percentage in the Atlantic spotted dolphin (37.5%; 3/8), followed by the striped dolphin (22.2%; 6/27), the Cuvier’s beaked whale (14.3%; 1/7), the common dolphin (12.5%; 1/8), and the bottlenose dolphin (4.8%; 1/21). Most of the HV-infected animals from our study were males (66.6%; 8/12): eight positive samples in 50 evaluated tissues (8/50; 16%) in males vs. 4/52 in females (7.7%), and juvenile (50%; 6/12).

Three sequences have been previously reported (case Nos. 1–3) (GenBank Acc. Nos. EU003440, KJ156330, and KJ156331. Ten unique HV nucleotide (nt) sequences of the partial DNA-polymerase gene (ranging from 153 to 213 bp in length, excluding primers) were obtained: case 4 (GenBank Acc. No. KY680657); case 5 (GenBank Acc. No. KY680656); case 6 (GenBank Acc. Nos. OM030214 and OM030215); case 7 (GenBank Acc. No. KY680659); Case 8 (GenBank Acc. No. KY680658); case 9 (GenBank Acc. No. MN179655); case 10 (GenBank Acc. No. MN179656); case 11 (GenBank Acc. No. MN179657), and case 12 (GenBank Acc. No. MN179658). Most of the sequences belonged to the Alphaherpesvirinae subfamily. Only one sequence (for reverse primer lecture) obtained from case 6 belonged to the Gammaherpesvirinae subfamily. CeMV was detected in three out 12 HV-positive animals from our study by one or more PCR methods (a, b and/or c): case 2 (a) [17], case 9 (b) [27], and case 12 (d) [27]. Flaviviruses were not detected in any of the HV-molecularly positive brain samples.

### 3.2. Histopathological Findings and Virus Strains

Histopathological evaluations of the CNS samples from these 12 HV-positive animals revealed perivascular cuffing in 10/12 (83.3%), meningitis in 9/12 (75%), microgliosis in 9/12 (75%), neuronal necrosis and associated focal neuronophagic nodules in 8/12 (66.6%), INIB in 6/12 (50%), malacia in 5/12 (41.6%), and haemorrhage in 5/12 (41.6%). Additional findings included choroiditis in 4/12 (33.3%). The inflammatory infiltrate was lymphoplasmacytic in all cases. The histopathological findings from each animal with herpesviral infection of the brain along with the corresponding GenBank Acc. Numbers of each obtained sequences, highest nucleotide identities, cycle threshold values and melting temperature from qPCR and immunohistochemical results are summarized in Table 2.

Case 1, an adult male bottlenose dolphin (172 nt sequence length), is a human alphaherpesvirus 1 (herpes simplex virus 1). Brain samples from this animal displayed 2/7 of the evaluated lesions: mild multifocal meningitis and perivascular cuffing.

Case 2, a subadult male striped dolphin, (194 nt sequence length) presented 4/7 of the evaluated lesions (meningitis, perivascular cuffing, microgliosis, and neuronal necrosis and associated focal neuronophagic nodules). The sequence obtained from this animal showed the highest nt similarity (99.45%) (82% Query Cover (QC)) with a sequence detected in the kidney of a striped dolphin stranded during the CeMV epizootic along the Mediterranean Spanish coast in 2007 (GenBank Acc. No. GQ888669). The animal from our study was also a striped dolphin co-infected with *dolphin morbillivirus* (DMV) stranded in 2007 in the Canary Islands.

**Figure 1:**
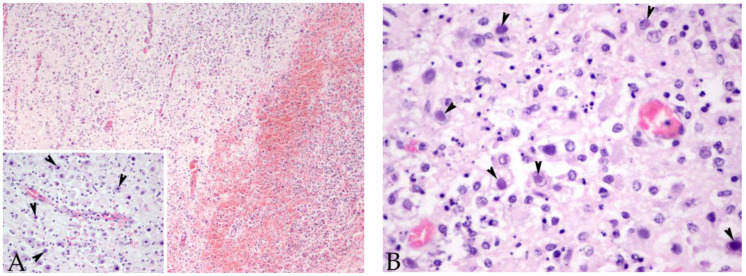
Brain cerebral cortex sample of case 4, a male striped dolphin calf presenting 7/7 of the evaluated brain lesions. (**A**) Suffusive haemorrhages and malacia are present. Original magnification ×10; haematoxylin and eosin staining. Inset: numerous basophilic intranuclear inclusion bodies were observed within the neurons and glial cells (arrowheads). Original magnification ×40; haematoxylin and eosin staining. (**B**) Higher magnification of the basophilic intranuclear inclusion bodies (arrowheads). Original magnification ×60; haematoxylin and eosin staining.

**Figure 2:**
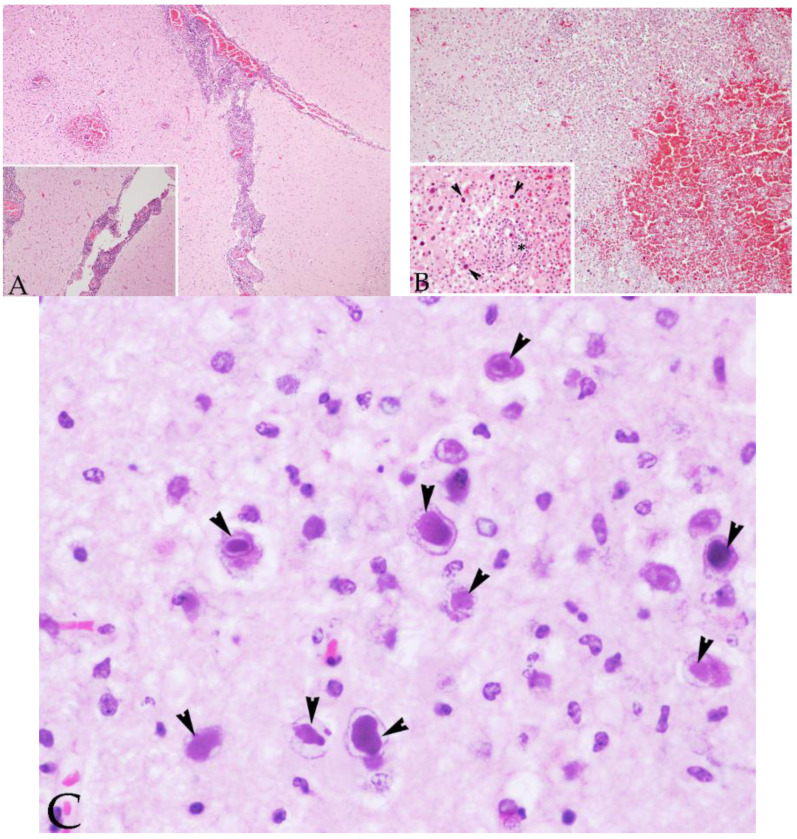
Histopathological brain lesions from case 5: (**A**) Brain cerebral cortex sample of case 5, a juvenile male striped dolphin presenting 7/7 of the evaluated brain lesions. Severe non-suppurative meningitis is present. Original magnification ×4; haematoxylin and eosin staining. Inset: Detail of perivascular cuffing and non-suppurative meningitis. Original magnification ×10; haematoxylin and eosin staining. (**B**) Brain cerebral cortex sample of case 5, a juvenile male striped dolphin presenting 7/7 of the evaluated brain lesions. Suffusive haemorrhages, malacia and perivascular cuffing are present. Original magnification ×10; haematoxylin and eosin staining. Inset: numerous amphophilic intranuclear inclusion bodies were observed within the neurons and glial cells (arrowheads). Detail of perivascular cuffing (asterisk). Original magnification ×40; haematoxylin and eosin staining. (**C**) Higher magnification of the basophilic intranuclear inclusion bodies (arrowheads). Original magnification ×60; haematoxylin and eosin staining.

Case 3, a subadult male striped dolphin (204 nt sequence length), showed the highest similarity (99.49–98.47%) (96–95% QC) with a sequence detected in a striped dolphin stranded in 2012 on the Portuguese coast (GenBank Acc. No. MG437217) and with three cases from our study (case Nos. 4, 5, and 12). Cases 3, 4, 5, and 12 displayed 7/7 of the evaluated lesions in the brain (Figure 1 and Figure 2), and in addition, case 5 presented non-suppurative choroiditis. Additionally, case 3 showed 100% nt identity (93% QC) with a sequence detected in the lung and the spleen of a beaked whale stranded in the Canary Islands in 2005 (GenBank Acc. No. GU066291).

A 203 nt sequence was obtained for case 4, a calf male striped dolphin, and a 214 nt sequence for case 5, a juvenile male striped dolphin.

**Figure 3:**
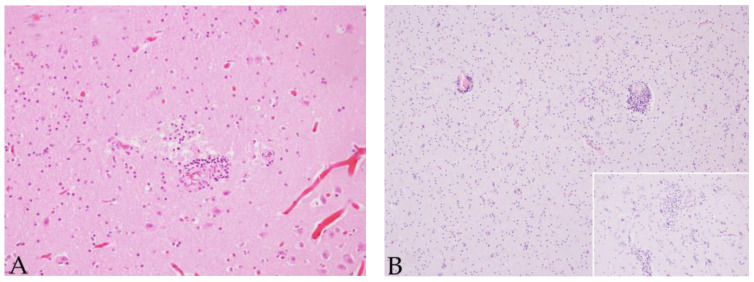
(**A**) Brain cerebral cortex sample of case 6, an adult female striped dolphin presenting 2/7 of the evaluated brain lesions. Mild lymphoplasmacytic perivascular cuffing are present. Original magnification ×20; haematoxylin and eosin staining. (**B**) Brain cerebral cortex sample of case 8, an adult male Atlantic spotted dolphin presenting 3/7 of the evaluated brain lesions. Diffuse microgliosis and moderate lymphoplasmacytic perivascular cuffing are present. Original magnification ×10; haematoxylin and eosin staining. Inset: Detail of a glial focus. Original magnification ×20; haematoxylin and eosin staining.

Two different sequences were obtained from case 6, an adult female striped dolphin. The sequence from the forward primer (153 nt sequence length) showed the highest nt similarity (93.46%) (100% QC) with α-HV sequences detected in the brain of a Risso’s dolphin (*Grampus griseus*) stranded in 2013 in the Mediterranean Sea (GenBank Acc. No. KP995683) and with the sequence obtained from the intestine of a striped dolphin stranded in the middle of the second Mediterranean CeMV epizootic (GenBank Acc. No. GQ888671). The reverse sequence (159 nt sequence length) showed the highest nt identity (99.38%) (100% QC) with a γ-HV sequence from a penile lesion of an adult free-ranging striped dolphin stranded in the Canary Islands in 2011 (GenBank Acc. No. KM248274) and 98.12% nt similarity (100% QC) with a pool of tissue samples (kidney and lung) from a common dolphin stranded on the Portuguese coast in 2011 (GenBank Acc. No. MG437207). Case 6 presented only 2/7 of the evaluated brain lesions: mild meningitis and perivascular cuffing (Figure 3A). Other findings included mild non-suppurative choroiditis.

Case 7, an adult male Cuvier’s beaked whale (nt sequence of 213 length), showed the highest nt similarity (90.37%) (87% QC) with a sequence detected in the kidney of a Blainville’s beaked whale (*Mesoplodon densirostris*) stranded in the central east Atlantic in 2005 (GenBank Acc. No. JN863234). None of the analysed lesions were observed in case 7. Case No. 8 (215 nt sequence length) (GenBank Acc. No. KY680658) showed the highest nt similarity (91.79%) (90% QC) with a sequence detected in the lung of a common dolphin stranded in Portugal in 2011 (GenBank Acc. No. MG437204) and 89.06% nt identity (88% QC) with buffy coat cells from a bottlenose dolphin (GenBank Acc. No. GQ429150).

Case 8, an adult male Atlantic spotted dolphin, displayed 3/7 of the analysed brain lesions (mild perivascular cuffing, microgliosis and multifocal microhaemorrhages) (Figure 3B) and non-suppurative choroiditis on histopathological examination. This animal also showed a systemic infection by *Staphylococcus aureus*.

Case 9, a juvenile female common dolphin (204 nt sequence length), showed the highest nt similarity (99.02% (100% QC) and 99.55 (95% QC)) with sequences detected in tissue pools from common dolphins stranded on the coasts of Portugal in 2011 (GenBank Acc. Nos. MG437212 and MG437210, respectively). Case 9 displayed 4/7 of the evaluated brain lesions: meningitis, perivascular cuffing, microgliosis and neuronal necrosis and associated focal neuronophagic nodules. This case was also co-infected with DMV.

**Figure 4:**
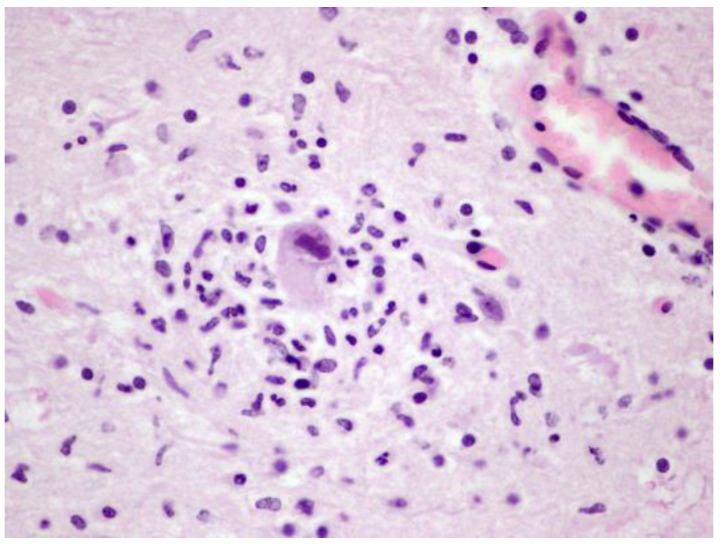
Brain cerebral cortex sample of case 10, a juvenile female striped dolphin presenting 5/7 of the evaluated brain lesions. A neuronophagic nodule surrounding a neuron with a basophilic intranuclear inclusion body is present. Original magnification ×60; haematoxylin and eosin staining.

Case 10, a juvenile female Atlantic spotted dolphin, (189 nt sequence length) showed the highest nt similarity (91.4%-90.21%) (100% QC) with the sequence detected in the brain of case 9 from our study and with sequences detected in common dolphins stranded on the coasts of Portugal in 2011 and 2012 (GenBank Acc. No. MG4337212, MG437211 (co-infected with DMV), MG437210 and MG437208). Case 10 displayed 5/7 of the evaluated brain lesions: meningitis, perivascular cuffing, microgliosis, INIB, neuronal necrosis and associated focal neuronophagic nodules (Figure 4).

**Figure 5:**
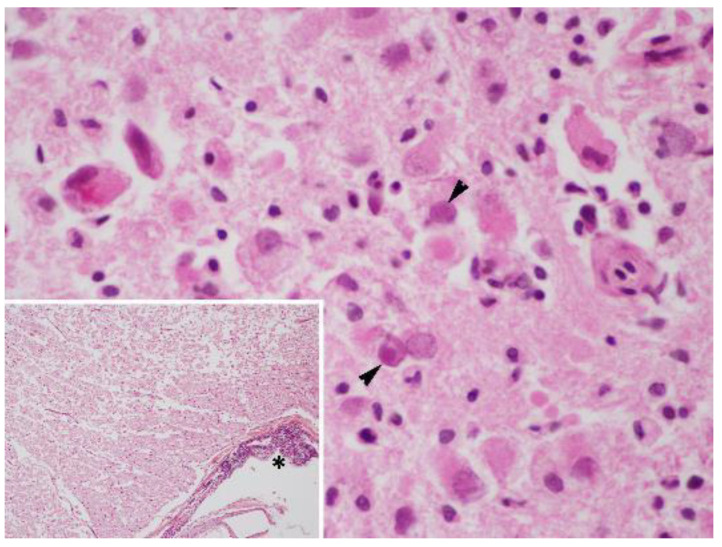
Brain cerebral cortex sample of case 11, a female of Atlantic spotted dolphin calf co-infected with Brucella sp., presenting 6/7 of the evaluated brain lesions. Multiple amphophilic intranuclear inclusion bodies within neurons (arrowheads) and satellitosis are present. Original magnification ×60; haematoxylin and eosin staining. Inset: medulla oblongata sample of case No 11. Moderate non-suppurative meningitis is present (asterisk). Original magnification ×10; haematoxylin and eosin staining.

Case 11, a calf female Atlantic spotted dolphin (nt sequence of 213 length) showed the highest nt similarity (92.56%) (100% QC) with a sequence detected in a pool of tissue samples from a common dolphin stranded on the coast of Portugal in 2011 (GenBank Acc. No. MG437213) and 92.09% nt identity (100% QC) with case 12 from our study. This animal displayed 6/7 of the evaluated brain lesions: meningitis, perivascular cuffing, microgliosis, INIB, malacia, and neuronal necrosis and associated focal neuronophagic nodules (Figure 5). *Brucella* spp. co-infection was suspected in this animal due to severe lymphohistiocytic meningitis affecting the brain and the spinal cord and confirmed by PCR.

**Figure 6:**
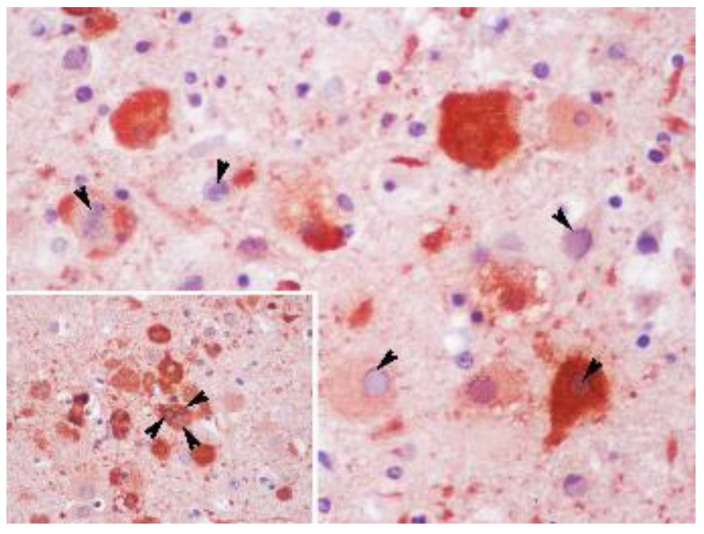
Brain cerebral cortex sample of case 12, a juvenile male striped dolphin presenting co-infection with DMV, presenting 7/7 of the evaluated brain lesions. Multiple basophilic intranuclear inclusion bodies within the neurons (arrowheads) can be seen with immunostaining against CDV. Original magnification ×60; immunohistochemistry against CDV counterstained with haematoxylin. Inset: Brain cerebral cortex sample of case 12. Details of basophilic intranuclear inclusion bodies within giant syncytial cells (arrowheads) are shown with immunostaining against CDV. Original magnification ×60; immunohistochemistry against CDV counterstained with haematoxylin.

Case 12, a juvenile male striped dolphin (213 nt sequence length), showed the highest similarity (99.06%) (99% QC) with case 5 from our study (GenBank Acc. No. KY680656) and 97.67% nt identity (100% QC) with a sequence detected in a striped dolphin co-infected with DMV stranded in the Portugal coast in 2012 (sc-221-2012) (GenBank Acc. No. MG437217). Case 12 was also co-infected with DMV, which sequence showed high identity with the DMV sequence detected in sc-221-2012. In both cases (case 12 and sc-221-2012), giant syncytial cells with amphophilic INIB were observed in the lymph nodes and lungs in the case from Portugal, and in the bran in the animal from our study. In contrast with the animal from Portugal, immunohistochemistry demonstrated the presence of morbillivirus antigens in neurons with INIB and in giant syncytial cells from the brain (Figure 6).

### 3.3. q-PCR Results

The limit of detection for the HV q-PCR was 1,1 copies. Different values for threshold cycles (Cts) were obtained in the q-PCR from the samples of our study (Table 2). The highest viral load was obtained from case 4 (Ct = 7.32) and the lowest from case 6 (Ct = 32.15). Ct values from 12.66 to 23.31 corresponded to cases 12, 7, 3, 10, 11 and 5, in which the melting temperature ranged from 86.5 °C to 91.5 °C (Table 2). Amplification curves were not obtained from cases 8 and 9, while the qPCR could not be performed in cases 1 and 2, as unfixed brain tissue samples were not available anymore. The conventional PCR was anew performed in available brain tissue samples (cases 3–12) with dissimilar results; at this time amplicons were obtained from all the cases, except for case 8.

### 3.4. Presence of HVs in Other Tissue Samples

Additionally, Herpesviral DNA was found in other tissue samples in five out twelve (5/12; 41.7%) brain HV-positive animals from our study (Table 3). In two cases, there was just another HV-positive tissue sample, while in the other three cases, there were two, three or five more HV-positive tissue samples. The lungs were positive in three cases, skin, mesenteric lymph node, liver, and the kidneys in two cases, respectively, and the spleen just in one case.

### 3.5. Immunohistochemical Findings

**Figure 7:**
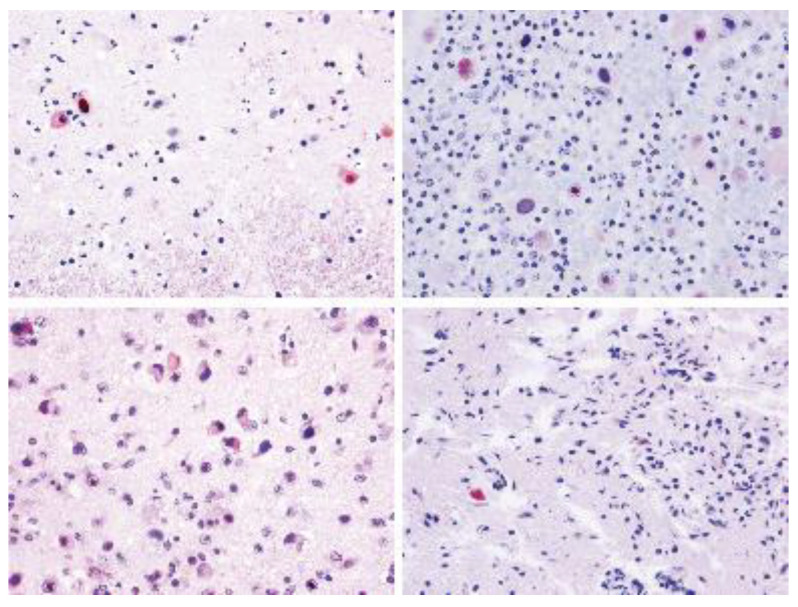
Immunohistochemistry of Anti-HSV1 in HV-positive brain tissue samples of cases 12, 5, 4, and 11. Inclusion bodies labelled immunopositively for herpesvirus antigen (red colour). Immunohistochemistry counterstaining with haematoxylin.

Positive immunolabeling with Anti-HSV1 antibody were obtained in 7 out 12 (58.3%) DNA herpesviral brain positive cases from our study (cases 3, 4, 5, 9, 10, 11 and 12). Immunostaining was mainly observed in the nucleus and cytoplasm of neurons and glial cells and INIBs, although unstained nuclear inclusions were frequently observed (Figure 7).

### 3.6. Phylogenetic Tree

**Figure 8:**
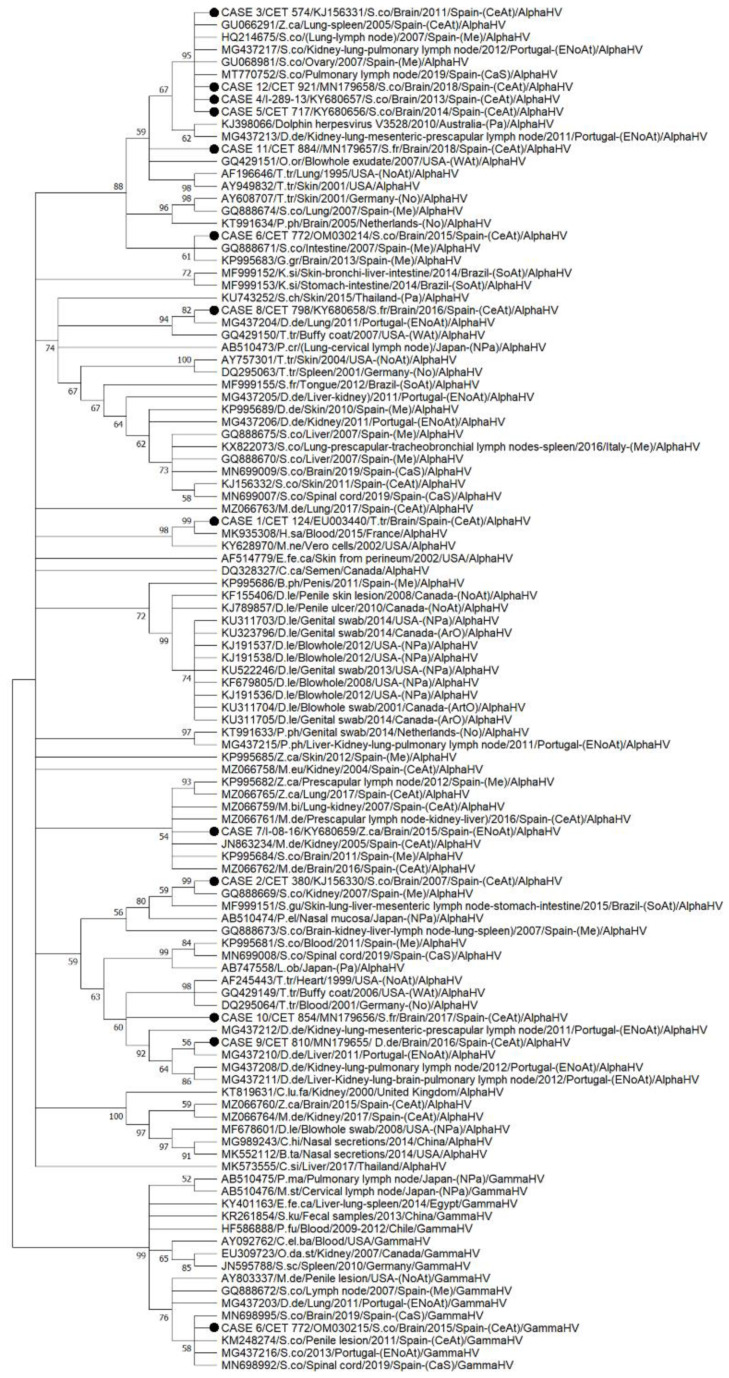
Nucleotide phylogenetic tree representing the relationships among cetaceans HVs. The phylogenetic tree was based on the herpesvirus DNA polymerase gene and constructed using the Tamura 3-parameter model (T92 + G) Maximum likelihood (ML), with an estimate of statistical support from 500 bootstrap replicates. Bootstrapping values are indicated as percentages next to the bifurcations (BVs less than 50 were collapsed into a polytomy). The name of each sequence includes the GenBank accession number (when available), cetacean species (B.ph, *Balaenoptera physalus*; D.le, *Delphinapterus leucas*; D.de, *Delphinus delphis*; G.gr, *Grampus griseus*; K.si, *Kogia sima*; L.ob, *Lagenorhynchus obliquidens*; M.de, *Mesoplodon densirostris*; O.or, *Orcinus orca*; P.cr, *Pseudorca crassidens*; P.el, *Peponocephala electra*; P.ph, *Phocoena phocoena*; S.ch, *Sousa chinensis*; S.co, *Stenella coeruleoalba*; S.fr, *Stenella frontalis*; S.gu, *Sotalia guianensis*; T.tr, *Tursiops truncatus*; and Z.ca, *Ziphius cavirostris*), the year, the geographic area of the stranding (BeS, Bering Sea; CEAt, Central Eastern Atlantic Ocean; In, Indian Ocean; Me, Mediterranean Sea; NEAt, Northeast Atlantic Ocean; Npa, North Pacific Ocean; NS, North Sea; NWAt, Northwest Atlantic Ocean; Pa, Pacific Ocean; SEAt, South Eastern Atlantic Ocean), and the tissues in which they were detected. Herpesviruses sequences from other species incorporated to the phylogenetic analysis included terrestrial and flying mammals (B.ta, *Bos taurus*; C.lu.fa, *Canis lupus familiaris*; C.hi, *Capra hircus*; C.ca, *Cervus canadensis*; C.el.ba, *Cervus elaphus barbarous*; E.fe.ca, *Equus ferus caballus*; H.sa, *Homo sapiens*; M.ne, *Macaca nemestrina*; O.da.st, *Ovis dalli stonei*; P.fu, *Pseudalopex fulvipes*; S.ku, *Scotophilus Kuhlii*; S.sc: *Sus scrofa*) and reptiles (C.si, *Crocodylus siamensis*).

The HV sequences from our study (*n* = 13) were placed in different branches within the nucleotide phylogenetic tree, although sequences from case Nos. 3, 4, 5 and 12 were within the same subclade, supported by a bootstrap value (BV) of 95%. The gammaherpesvirus sequence from case 6 was clustered with a sequence detected in the penile lesion of a striped dolphin stranded in the Canary Islands in 2011 (Figure 8).

## 4. Discussion

Herpesviral DNA was detected in 11.6% of the analysed brain samples from five different cetaceans’ species stranded in the Central East Atlantic (Canary Islands, Spain), which is higher to that reported in harbour porpoises from the Netherlands (6.7%) [11]. However, higher frequencies of HVs have been reported in cetaceans stranded in the Mediterranean and the Cantabrian Sea, which may be related to impairment of their immune system activity in response to potential synergistic factors, such as chemical contamination: the Mediterranean Sea is one of the most polluted seas worldwide [48], chronic stress, and/or infection with viral agents, such as Morbillivirus [49,50]. Most of the massive die-off of cetaceans related to morbillivirus infection (MI) occurred in the Mediterranean Sea [51]. Herpesvirus was detected in 62.5% (5/8) of DMV-molecularly positive striped dolphins stranded during the cetacean morbillivirus epizootic along the Mediterranean Spanish coast in 2007 [28]. Herpesvirus and morbillivirus co-infection was detected in 25% (3/12) of the animals from our study, in 23, 7% (9/38) of the animals stranded in Valencia [30], in 14, 2% (2/14) of animals stranded in the coasts of Portugal [19] and in 0% (0/11) of the striped dolphins stranded in Cantabria [29]. Herpesvirus infection can be acquired during the acute and subacute phases of the MI because of the severe lymphoid depletion induced by these viruses. The morbilliviruses are cleared from the animals overcoming the MI, while present herpesviruses may lay dormant for long periods and reactivate later in life, being molecularly detectable in both situations. Thus, the detection of HVs without the detection of morbillivirus does not necessarily imply the non-implication of morbilliviruses in contracting herpesvirus infection. This could explain the absence of MI in the analysed striped dolphins from the Cantabrian Sea, although other factors involved in this higher HV infection occurrence could be related to the number of analysed tissue samples; as the average number of samples per animal increases, a higher percentage of HV-positive samples is found (Table 4).

Regarding HV frequencies in different cetaceans’ species, it is noteworthy that despite the high incidence of HV infection in the Atlantic spotted dolphin species in our study, there is only one previous description of HV infection in this species; specifically, an α-HV was detected in a lingual sample of a dolphin from Brazil [13]. Therefore, this study reported the first detection of α-HVs from the brain in the Atlantic spotted dolphin species. Concerning striped dolphins, HV infection has been frequently reported, with detections mainly in the Mediterranean Sea [16,28,29,30,31,52] and less frequently in the north and central Atlantic [17,19]. The presence of HVs in Cuvier’s beaked whales has been rarely reported, with only three previous cases in the Atlantic and the Mediterranean [8,30,31,32]. The detection of HVs in the common dolphin is quite recent, with 10 positive cases for viral infection found on the coasts of Portugal [19]. The presence of HVs in the bottlenose dolphin species has been reported relatively often in the western North Atlantic [7,18,53] and rarely in the Mediterranean [30,31].

Regarding sex influence in HV infection frequency, most of the infected cetaceans in our study were males. Similarly, a higher percentage of males than females was observed when brain samples of harbour porpoises from the Netherlands were analysed (80%; *n* = 5) [11]. The prevalence was also higher in males in a striped dolphin mass stranding in Cantabria (Spain) (9/11, 81.82%) [29], and in two studies carried out in Brazil and Japan (2/3 HV-positive animals with identified sex were males; 66.67%) [12,13]. No sex predisposition was found in a systematic survey on the coast of Portugal (*n* = 179) [19]. However, a higher percentage of female HV-positive tissues than male HV-positive tissues was observed in a systematic survey on the Mediterranean coast of Spain, particularly in the nervous, urinary, endocrine, and reproductive systems (Valencia) [29,30] and in a retrospective HVs surveillance in beaked whales stranded in the Canary Islands (5/8, 62.5%) [32]. This discrepancy in sex predisposition has not been previously elucidated and further studies (with a greater number of animals) would be necessary to determine if sex can act as a risk factor for HV infection. In addition, in five of the eight males from our study (5/8; 62.5%) the virus was detected in several of the analysed organs (from 2 to 6, depending on the individual) in contrast to females (0/4; 0%).

A higher percentage of HV infection was found in the juvenile-subadult group in our study than the rest of the age groups. Similarly, all of the harbour porpoises with the presence of herpesviral DNA in the brain were juveniles [11]. In addition, an age-group predisposition was found in a systematic survey carried out on the coast of Valencia (Mediterranean, Spain), in which a lower percentage was observed in suckling calves and neonates than in juveniles, in a similar way to our study [30,31]. It has been demonstrated that the immune status alone or in combination with the age of the specimen can affect the course of an infection [1].

Despite the increasing number of reported HV infections in cetaceans, there is still a gap in the knowledge about its pathogenicity and contribution to the cause of death. In this study, we tried to correlate brain lesions with the detected viral strain in each case. Much of the brain HV sequences obtained in this study belonged to the α-herpesvirinae subfamily, as it was only in one case (case 6) that the detected sequence was included in the γ-herpesvirinae subfamily. γ-HVs are more frequently detected in genital and mucosal lesions [7,11,13,19,20,21,22,23,24,29,30] and, accordingly, the γ-HV detected in our study showed the highest nt identity with a γ-HV sequence detected in the penile lesion of an adult free-ranging striped dolphin stranded in the Canary Islands in 2011 [23]. This could suggest that the virus may reach the brain through autonomic neurons in the genitals, as has previously been proposed [29], although other proposed mechanisms for viruses neuroinvasion is through infected monocytes or macrophages that infiltrate the brain through the blood–brain barrier. [54]

Although it is well known that γ-HVs mainly infect and establish latency in lymphocytes, there is accumulating scientific evidence suggesting the possible neurotropism of human gammaherpesviruses [55,56,57] and their involvement in various neurological diseases [58,59,60]. Recently, the presence of several γ-HVs in brain tissue samples from odontocete cetaceans stranded in the Mediterranean (*n* = 8) and the Cantabrian (*n* = 4) seas with non-specific associated brain lesions (mild to severe lymphoplasmacytic and hystiocytic meningoencephalitis) has been reported [29,30]. In addition, the first γ-HV brain infection in cetaceans was reported in a female common minke whale calf (*Balaenoptera acutorostrata*) stranded on the Mediterranean coast of Valencia [15]. The lack of pathological description in some of these cases and the potential presence of other agents (i.e., *Brucella* spp. and *Toxoplasma gondii*) that could have participated in the pathogenesis of such lesions cannot been ruled out, making it difficult to interpret the role of γ-HVs as a causative agent of brain damage in cetaceans. In our study, lesions were minimal, and no co-infection was detected in case 6, although two different sequences of HVs were detected in the brain sample, with the others included in the α-herpesvirinae subfamily. The detection of different viral strains of HVs in cetaceans has been previously reported [7,17,19,28,32].

Mild to moderate lymphoplasmacytic inflammation within the CNS, as described in most cetaceans in which a- and g-HVs have been molecularly detected, could be non-specific and difficult associate with any specific etiological agent, even more in cases of co-infection [11,17,27,28]. In our study, brain lesions severe enough to induce clinical manifestations leading to death and/or stranding were found in seven animals. In addition, immunohistochemical results were in accordance with histopathological findings, as most of the animals presenting acute lesions, such as INIBs, malacia and neuronal necrosis with associated focal neurophagic nodules were positive for Anti-HSV1 in brain tissue samples, confirming the role of these viruses as an etiologic agent of such lesions. In contrast, no viral antigen was detected in those animals with signs of chronic active encephalitis, consisting of meningeal and/or perivascular infiltration of lymphocytes and gliosis. Previous studies in humans described that in patients with chronic inflammation associated with herpes simplex encephalitis, the viral antigen could not be detected by immunohistochemical methods, though specific viral DNA was demonstrated by PCR, suggesting the persistence of the virus [61]. The only exception was case 2, which presented neuronal necrosis with associated focal neurophagic nodules, although these lesions could also be attributable to the DMV co-infection detected in this animal [17,27].

Five co-infections (41.6%; 5/12) were detected in the current study. Fatal *Staphylococcus aureus* septicaemia was diagnosed (bacterial culture) in case 8, an adult male Atlantic spotted dolphin with pyogranulomatous myocarditis, bronchopneumonia, adrenalitis, nephritis and lymphadenitis [27]. *Brucella* sp. infection of the CNS was diagnosed by PCR in case 11, a female Atlantic spotted dolphin calf. In case 11, some of the observed brain lesions, particularly meningitis and perivascular cuffing, could be attributed to this pathogen rather than to HVs [27]. HV and DMV co-infection was present in Cases 2, 9 and 12. Co-infections of morbilliviruses and herpesviruses have been reported since the first seal morbillivirus epidemic occurred in 1988 [62]. As in our study (cases. 2, 9 and 12), previous descriptions reported the presence of both pathogens in the brain of stranded cetaceans [28,30]. Case 2 was stranded in 2007 in the Canary Islands in the midst of the Mediterranean outbreak, and the DMV sequence obtained from this case formed a subclade with all the published sequences obtained during the 2006–2008 Mediterranean epizootic [17], suggesting inter-animal virus transmission in the Strait of Gibraltar between these two populations of striped dolphins, as previously suggested [63]. The high homology between the HV sequence from case 2 with a sequence detected in a striped dolphin stranded during the DMV epizootic along the Mediterranean Spanish coast in 2006–2008 [28] reinforces this hypothesis and suggests a possible relationship between these two viruses, according to the epidemiology of morbillivirus infection. This hypothesis is also reinforced by case 12, a striped dolphin stranded in the Canary Islands in 2018, in which the sequences for both DMV (unpublished data) and HV showed the highest homology with sequences detected in one animal from the same species, a striped dolphin stranded in Portugal in 2012 (sc-221-2012) [19,64]. In both cases, (case 12 and sc-221-2012), the presence of giant syncytial cells (immunostaining against morbillivirus in case 12) with amphophilic INIB could be interpreted as cell superinfection, a rare phenomenon in which a cell previously infected by one virus becomes co-infected with a different strain of the same virus (homologous recombination) or another virus (heterologous recombination) [65]. This is a common mechanism for some viruses, while for others (including α-HVs), superinfection is rare due to the phenomenon known as superinfection exclusion (the first virus infecting a cell prevents subsequent viruses from further infecting the same cell) [66]. Further analyses are required to assess the significance of this outstanding finding. In case 8, a common dolphin stranded in the Canary Islands in 2016, we observed a similar phenomenon; HV sequences detected in this case showed the highest similarity with sequences detected in two specimens from the same species stranded on the Portuguese coast in 2011, and the DMV sequence (unpublished data) showed the highest homology with a sequence detected in a striped dolphin stranded in the Mediterranean Sea (Italy) in the same year (2011). DMV was associated with an unusual mass mortality episode in striped dolphins in the Western Mediterranean Sea in 2011 [67]. HV co-infection with DMV may suggest that virulence of herpesviral infection was dependent upon the immunosuppression caused by the virus, as has been previously described for two DMV-positive striped dolphins stranded during the cetacean morbillivirus epizootic along the Mediterranean Spanish coast in 2007 and co-infected with HVs [16,28].

The HV sequences detected herein were in different branches in the phylogenetic tree, although only six sequences were in clades supported by BV greater than 80% (indicating strong support for these clades). Four sequences, from Case Nos. 3, 4, 5 and 12, were in a clade supported by a BV of 95. All the sequences from our study included in this branch were from striped dolphins that displayed 100% (7/7) of the evaluated brain lesions, including malacia, haemorrhages and INIBs, and were categorized as juveniles (3/4) or calves (1/4). A correlation between the severity of the herpesvirus infection and decreased immune competence, as could occur in immature animals due to loss of maternal immunity, has been previously reported [68]. All the animals included in this clade (*n* = 9), except for a Cuvier’s beaked whale, were striped dolphins. Most of the sequences included in this cluster (7/9; 77.8%) have been previously associated with pathological findings, consisting of severe cell necrosis and/or the presence of amphophilic INIB in their respective tissues [8,16,19] Further studies should analyse whether these α-HV variants could represent a pathogenic viral strain in cetaceans. One sequence (case 8; Atlantic spotted dolphin) was clustered (82% BV) with a sequence detected in the lung of a common dolphin stranded in Portugal in 2011 [64]; and a sequence from case 2 (striped dolphin) was clustered (99% BV) with a sequence detected in the kidney of another striped dolphin stranded in the Mediterranean Sea in 2007 [28]. Both cases were stranded in the same year and were co-infected with DMV. Further research is needed to understand the interaction between these viruses over the course of their infection and transmission.

The role of viral factors in the course of infection is determined by both viral and host factors [69]. Co-infections [1], and/or other pathogenic factors such as immuno-(in)competence of the natural hosts [3,70], could also be involved in the severity of the observed brain lesions in some of the cases from our study. Higher virus load was in accordance with lesions severity in only some cases (cases 4, 12, and 3); a higher value was also detected in case 7, in which no lesions were found. Two samples could not be amplified with this adapted universal nested qPCR. This could be due to DNA degradation, although the possibility of human error cannot be excluded as an amplicon was obtained from one of them when a simultaneous conventional PCR was carried out.

## 5. Conclusions

Several studies have reported the presence of α- and γ-HVs in cetacean brains: most of them with non-specific associated lesions, mainly lymphoplasmacytic chronic inflammation, and without tissue identification of the virus by immunohistochemistry or transmission electron microscopy. Here, we present serious brain injuries molecularly and immunohistochemically proven to be caused by different variants of HVs in seven out twelve of the animals. The seriousness of brain injuries could lead them to stranding and/or death. Some pathogenic factors, such as the age of the animals, the presence of co-infections, and/or the viral strain, should be considered when assessing the severity of the observed brain lesions. Almost identical a-HVs sequences were detected in four animals with the most severe lesions, suggesting a putative pathogenic herpesvirus variant.

## Figures and Tables

**Table 1:** Summarized data from the 12 animals with herpesvirus brain infections. F, female; M, male; AC, age class; A, adult, J, juvenile; C, calf; SD, stranding date; SP, stranding place; CC, carcass condition.

Case No.	Lab Reference	Species	Sex	AC	SD	SP	CC
1	I-086/01 (CET 124)	*Tursiops truncatus*	M	A	11/04/2001	Tenerife (Canary Islands)	Fresh
2	I-091/07 (CET 380)	*Stenella coeruleoalba*	M	J	16/04/2007	Tenerife (Canary Islands)	Fresh
3	I-145/11 (CET 574)	*Stenella coeruleoalba*	M	J	01/05/2011	Gran Canaria (Canary Islands)	Fresh
4	I-289/13 (NA)	*Stenella coeruleoalba*	M	C	31/08/2013	Cádiz (Andalusia)	Moderate autolysis
5	I-151/14 (CET 717)	*Stenella coeruleoalba*	M	J	21/05/2014	Gran Canaria (Canary Islands)	Very fresh
6	I-416/15 (CET 772)	*Stenella coeruleoalba*	F	A	21/08/2015	Lanzarote (Canary Islands)	Fresh
7	I-08/16 (NA)	*Ziphius cavirostris*	M	A	02/12/2015	Huelva (Andalusia)	Moderate autolysis
8	I-287/16 (CET 798)	*Stenella frontalis*	M	A	08/04/2016	Tenerife (Canary Islands)	Fresh
9	I-907/16 (CET 810)	*Delphinus delphis*	F	J	03/07/2016	Tenerife (Canary Islands)	Fresh
10	I-167/17 (CET 854)	*Stenella frontalis*	F	J	15/05/2017	Tenerife (Canary Islands)	Moderate autolysis
11	SA038/18 (CET 884)	*Stenella frontalis*	F	C	16/01/2018	Tenerife (Canary Islands)	Fresh
12	SA223/18 (CET 921)	*Stenella coeruleoalba*	M	J	05/07/2018	Gran Canaria (Canary Islands)	Moderate autolysis

**Table 2:** Classical central nervous system (CNS) lesions associated with the presence of α-HVs consisted of meningeal mononuclear cell infiltrates (Me), lymphoplasmacytic perivascular cuffs (PC), diffuse microgliosis (M), intranuclear inclusion bodies (INIB), Malacia (Ma), neuronal necrosis and associated focal neurophagic nodules (NNs), and haemorrhages (H). OL: other lesions; C-I: co-infections; N.I.: nucleotide identity (with highest query cover); HSV-1: herpes simplex human type 1; α: alphaherpesvirus; γ: gammaherpesvirus; qPCR: quantitative polymerase chain reaction; Ct: threshold cycle; mTª: melting temperature; IHQ: immunohistochemistry; NA: not applicable. Highest nucleotide identities between sequences from our study are indicated in boldface.

C. N°	CNS Lesions									GenBank Acc. No.	N.I.	qPCR	qPCR	IHQ
	Me	PC	M	INIB	Ma	NNs	H	OL	C-I			Ct	mTª	
1	-	X	X	-	-	-	-	-	-	EU003440	95.79% MN401208 (HSV-1)	NA	NA	-
2	X	X	X	-	-	X	-	-	DMV	KJ156330	99.45% GQ888669 (α)	NA	NA	-
3	X	X	X	X	X	X	X	-	-	KJ156331	**99.49% KY680657**(α)	17.74	91	+
4	X	X	X	X	X	X	X	-	-	KY680657	**100% KY680656**(α)	7.32	90.50	+
5	X	X	X	X	X	X	X	Choroiditis	-	KY680656	**99.06% MN179658**(α)	23.31	90.00	+
6	X	X	-	-	-	-	-	Choroiditis	-	OM030214 OM030215	93.46% KP995683 (α)99.38% KM248274 (γ)	32.15	88.00	-
7	-	-	-	-	-	-	-	-	-	KY680659	90.37% JN863234 (α)	12.91	86.50	-
8	-	X	X	-	-	-	X	Choroiditis	*S. aureus*	KY680658	91.79% MG437204 (α)	-	-	-
9	X	X	X	-	-	X	-	-	DMV	MN179655	99.02% MG437212 (α)	-	-	+
10	X	X	X	X	-	X	-	-	-	MN179656	91.4% MG4337212 (α)	22.20	91.50	+
11	X	X	X	X	X	X	-	-	*Brucella spp*.	MN179657	92.56% MG437213 (α)	22.81	90.50	+
12	X	X	X	X	X	X	X	Choroiditis, syncytia	DMV	MN179658	**99.06% KY680656**(α)	12.66	90.50	+

**Table 3:** Tissue samples tested for HV in the twelve animals with HV-molecularly brain samples from our study. Positive samples are indicated in boldface.

Case No.	Tissue Samples Analysed for HV in Animals with HV-Molecularly Positive Brains
1	Skeletal muscle, **lung**, liver, kidney, **brain**
2	Skin, skeletal muscle, lung, liver, kidney, **brain**, spleen
3	**Skin**, skeletal muscle, lung, liver, mesenteric lymph node, kidney, **brain**, spleen
4	**Skin**, skeletal muscle, **lung**, liver, **mesenteric lymph node**, kidney, **brain**, spleen
5	Skin, skeletal muscle, lung, liver, mesenteric lymph node, kidney, **brain**, spleen
6	Skin, skeletal muscle, lung, mesenteric lymph node, kidney, **brain**, spleen
7	**liver**, **kidney**, **brain**
8	lung, prescapular lymph node, intestine, **brain**, spleen
9	lung, prescapular lymph node, heart, intestine, **brain**, spleen
10	lung, prescapular lymph node, intestine, **brain**, spleen
11	Skin, skeletal muscle, lung, liver, mesenteric lymph node, kidney, **brain**, spleen
12	Skin, skeletal muscle, **lung**, **liver**, **mesenteric lymph node**, **kidney**, **brain**, **spleen**

**Table 4:** Representative comparative scheme between different HV-infection frequencies related to the number of tissue samples and animals analysed. 13 *: this is the number of additional HV-positive animals not included in the present analysis of herpesviral encephalitis in cetaceans.

Average Samples per Analysed Animals	Positive Animals	Positive Tissues	HV-Positive Animals (%)	HV-Positive Tissue Samples (%)	References
178/79; 2.25	4	9	5.06%	5%	Miyoshi et al., 2011 [12]
446/103; 4.33	12 + 13 * = 25	40	24.3%	8.9%	Our study
294/55; 5.34	8	15	14.5%	5.1%	Felipe-Jiménez et al., 2021 [32]
182/14; 13	11	34	78.6%	18.68%	Vargas Castro et al., 2020 [29]
966/47; 20.25	38	121	80.85%	12.5%	Vargas-Castro et al., 2021 [30]

## Data Availability

Data are contained within the article.

## Supplementary Materials

The following supporting information can be downloaded at: https://www.mdpi.com/article/10.3390/ani12091149/s1, Table S1. Anamnestic and stranding data of animals under study.
