# Supplementary material for: Molecular Characterization of Herpesviral Encephalitis in Cetaceans: Correlation with Histopathological and Immunohistochemical Findings
Source: Animals (Basel). 2022 Apr 29;12(9):1149. doi: 10.3390/ani12091149 (PMC9105563; doi:10.3390/ani12091149)
Supplement: Supplementary file 1 [file animals-12-01149-s001.zip › animals-1685797-supplementary.pdf]

Supplementary Material Table S1. Anamnestic and stranding data of animals under study.

ID	Sp	Sex	AC	DS	ST	SP	CC
508/96 (CET 34)	*Gma*	M	Juvenile	28/07/1996	Alive	Tenerife	2
290/97 (CET 42)	*Gma*	M	Adult	ND	Dead	ND	2
177/99 (CET 59)	*Gma*	F	Adult	09/04/1999	Alive	Gran Canaria	2
323/99 (CET 78)	*Ttr*	M	ND	02/09/1999	Alive	Gran Canaria	1
044/00 (CET 93)	*Ttr*	M	Juvenile	ND	Dead	Tenerife	2
048/00 (CET 94)	*Ttr*	M	Juvenile	ND	Dead	Tenerife	2
060/00 (CET 96)	*Gma*	F	Adult	22/01/2000	Alive	Gran Canaria	4
061/00 (CET 97)	*Sco*	M	ND	30/01/2000	Alive	Fuerteventura	1
175/00 (CET 99)	*Ttr*	F	Juvenile	16/03/2000	Dead	Gran Canaria	3
217/00 (CET 100)	*Sbr*	M	Adult	06/04/2000	Dead	Tenerife	2
258/00 (CET 103)	*Zca*	M	Juvenile	19/04/2000	Dead	Fuerteventura	3
339/00 (CET 110)	*Dde*	F	Adult	13/06/2000	Alive	Gran Canaria	1
**I-086/01 (CET 124)**	*Ttr*	M	Adult	11/04/2001	Dead	Tenerife	2
I-244/01 (CET 133)	*Gma*	M	Calf	24/06/2001	Dead	Tenerife	2
I-245/01 (CET 134)	*Meu*	F	Calf	28/06/2001	Alive	Gran Canaria	1
I-267/01 (CET 139)	*Pma*	F	Juvenile	21/08/2001	Dead	Tenerife	3
I-300/01 (CET 145)	*Ttr*	F	Adult	03/10/2001	Alive	Gran Canaria	1
I-359/01 (CET 150)	*Gma*	F	Adult	08/12/2001	Dead	Tenerife	2
I-064/02 (CET 160)	*Sco*	M	Adult	28/03/2002	Dead	Fuerteventura	2
I-083/02 (CET 168)	*Ttr*	M	Juvenile	11/05/2002	Dead	Tenerife	2
I-088/02 (CET 171)	*Ttr*	F	Juvenile	30/05/2002	Dead	Tenerife	2
I-109/02 (CET 178)	*Sco*	M	Juvenile	19/08/2002	Alive	Gran Canaria	2
I-117/02 (CET 181)	*Zca*	M	Juvenile	24/09/2002	Alive	Fuerteventura	2
I-118/02 (CET 182)	*Zca*	M	Juvenile	24/09/2002	Dead	Fuerteventura	2
I-119/02 (CET 183)	*Zca*	M	Juvenile	24/09/2002	Dead	Fuerteventura	2
I-120/02 (CET 184)	*Zca*	M	Juvenile	24/09/2002	Dead	Fuerteventura	2
I-121/02 (CET 185)	*Meu*	F	Adult	24/09/2002	Dead	Fuerteventura	2
I-154/02 (CET 195)	*Sco*	M	Adult	13/11/2002	Alive	Gran Canaria	1
I-002/03 (CET 197)	*Sfr*	M	Adult	04/01/2003	Dead	Tenerife	3
I-006/03 (CET 199	*Ggr*	M	Adult	19/01/2003	Dead	Gran Canaria	3
I-128/03 (CET 209)	*Ttr*	F	Juvenile	15/05/2003	Dead	Gran Canaria	3
I-238/03 (CET 213)	*Mde*	F	Adult	28/06/2003	Alive	Gran Canaria	1
I-034/04 (CET 236)	*Zca*	F	Calf	21/03/2004	Dead	La Graciosa	3
I-127/04 (CET 259)	*Meu*	F	Juvenile	21/06/2004	Dead	Fuerteventura	2
I-130/04 (CET 260)	*Sco*	F	Juvenile	27/06/2004	Alive	Fuerteventura	2
I-032/05 (CET 288)	*Sco*	F	Adult	07/03/2005	Dead	Fuerteventura	2
I-225/05 (CET 305)	*Ttr*	F	Juvenile	18/07/2005	Alive	Lanzarote	1
I-265/05 (CET 308)	*Sco*	F	Juvenile	16/08/2005	Alive	Gran Canaria	2
I-445/05 (CET 314)	*Ttr*	F	Juvenile	28/10/2005	Dead	Gran Canaria	3
I-132/06 (CET 333)	*Meu*	F	Juvenile	28/03/2006	Alive	El Hierro	2
I-133/06 (CET 334)	*Meu*	F	Juvenile	28/03/2006	Alive	El Hierro	2
I-141/06 (CET 338)	*Meu*	F	Juvenile	06/04/2006	Dead	Gran Canaria	2
I-086/07 (CET 363)	*Sfr*	F	Juvenile	01/01/2007	Dead	Lanzarote	2
I-071/07 (CET 373)	*Dde*	F	Adult	26/03/2007	Alive	La Graciosa	2
**I-091/07 (CET 380)**	*Sco*	M	Juvenile	16/04/2007	Dead	Tenerife	2
I-318/07 (CET 400)	*Sco*	M	Adult	25/09/2007	Dead	Fuerteventura	2
I-088/08 (CET 407)	*Ttr*	F	Adult	18/01/2008	Dead	Tenerife	2
I-095/08 (CET 412)	*Sco*	M	Calf	10/03/2008	Alive	Gran Canaria	2

I-133/08 (CET 420)	*Ttr*	F	Adult	29/03/2008	Dead	Gran Canaria	3
I-223/08 (CET 429)	*Sco*	F	Juvenile	12/04/2008	Dead	Fuerteventura	2
I-149/08 (CET 431)	*Ggr*	M	Juvenile	20/04/2008	Alive	Tenerife	2
I-208/08 (CET 450)	*Ttr*	F	Juvenile	13/05/2008	Dead	Tenerife	2
I-214/08 (CET 451)	*Sfr*	ND	Calf	15/05/2008	Dead	Gran Canaria	4
I-237/08 (CET 456)	*Ggr*	F	Adult	17/06/2008	Alive	Gran Canaria	2
I-320/08 (CET 472)	*Ggr*	F	Calf	07/11/2008	Dead	Fuerteventura	2
I-343/08 (CET 473)	*Sbr*	M	Adult	18/11/2008	Alive	Gran Canaria	2
I-001/09 (CET 474)	*Sco*	M	Adult	31/12/2008	Dead	Lanzarote	2
I-007/09 (CET 476)	*Sco*	F	Adult	22/01/2009	Dead	Fuerteventura	2
I-051/09 (CET 483)	*Ggr*	M	Adult	06/03/2009	Dead	Fuerteventura	2
I-169/09 (CET 502)	*Sco*	F	Juvenile	22/07/2009	Alive	Lanzarote	1
I-296/09 (CET 509)	*Ttr*	M	Juvenile	09/12/2009	Dead	Tenerife	3
I-117/10 (CET 526)	*Ttr*	F	Adult	29/03/2010	Dead	Tenerife	2
I-123/10 (CET 530)	*Sfr*	F	Adult	13/04/2010	Dead	Tenerife	2
I-132/10 (CET 533)	*Ggr*	M	Adult	20/04/2010	Dead	Fuerteventura	3
I-136/10 (CET 534)	*Ggr*	M	Juvenile	22/04/2010	Alive	Tenerife	2
I-212/10 (CET 543)	*Ttr*	M	Adult	02/07/2010	Dead	Lanzarote	3
I-284/10 (CET 549)	*Ggr*	F	Adult	17/09/2010	Dead	Tenerife	2
**I-033/11 (CET 554)**	*Sco*	F	Adult	21/12/2010	Alive	Gran Canaria	1
I-014/11 (CET 558)	*Sco*	F	Adult	10/02/2011	Dead	Lanzarote	2
I-083/11 (CET 564)	*Ttr*	M	Adult	22/03/2011	Dead	Lanzarote	2
I-093/11 (CET 566)	*Sco*	F	Adult	26/03/2011	Dead	Tenerife	2
I-102/11 (CET 571)	*Gma*	F	Adult	01/04/2011	Dead	Gran Canaria	3
**I-145/11 (CET 574)**	*Sco*	M	Juvenile	01/05/2011	Alive	Gran Canaria	2
I-171/11 (CET 577)	*Sco*	M	Adult	16/05/2011	Alive	Tenerife	2
I-183/11 (CET 578)	*Ggr*	F	Adult	29/05/2011	Alive	La Gomera	2
I-192/11 (CET 580)	*Dde*	M	Adult	04/07/2011	Dead	Fuerteventura	2
I-366/11 (CET 592)	*Ttr*	F	Calf	05/11/2011	Dead	La Gomera	3
I-72/12 (CET 613)	*Dde*	M	Adult	27/04/2012	Dead	Gran Canaria	2
I-131/12 (CET 631)	*Meu*	M	Adult	21/10/2012	Dead	Fuerteventura	3
I-073/13 (CET 668)	*Dde*	F	Adult	22/05/2013	Alive	Tenerife	2
**I-289/13 (NA)**	*Sco*	M	Calf	31/08/2013	Dead	Cádiz	3
I-149/14 (CET 716)	*Sco*	M	Juvenile	19/05/2014	Dead	La Graciosa	3
**I-151/14 (CET 717)**	*Sco*	M	Juvenile	21/05/2014	Alive	Gran Canaria	1
I-317/14 (CET 732)	*Sco*	F	Juvenile	11/11/2014	Dead	Gran Canaria	2
I-342/14 (CET 734)	*Kbr*	M	Adult	20/11/2014	Dead	Fuerteventura	2
I-351/14 (CET 737)	*Kbr*	F	Juvenile	09/12/2014	Dead	Fuerteventura	3
I-196/15 (CET 746)	*Gma*	F	Adult	25/02/2015	Dead	Lanzarote	3
I-249/15 (CET 751)	*Ggr*	F	Adult	16/03/2015	Dead	Tenerife	2
I-280/15 (CET 758)	*Gma*	M	Calf	15/05/2015	Dead	Fuerteventura	3
I-284/15 (CET 760)	*Gma*	M	Adult	20/05/2015	Dead	Tenerife	3
I-384/15 (CET 767)	*Dde*	F	Adult	07/07/2015	Dead	Fuerteventura	2
**I-416/15 (CET 772)**	*Sco*	F	Adult	21/08/2015	Dead	Lanzarote	2
I-461/15 (CET 776)	*Ttr*	M	Juvenile	24/09/2015	Alive	Tenerife	2
**I-08/16 (NA)**	*Zca*	M	Adult	02/21/2016	Dead	Huelva	3
I-174/16 (CET 790)	*Sco*	M	Adult	09/03/2016	Dead	Lanzarote	2
I-286/16 (CET 797)	*Sfr*	F	Adult	07/04/2016	Dead	Tenerife	2
**I-287/16 (CET 798)**	*Sfr*	M	Adult	08/04/2016	Dead	Tenerife	2
I-600/16 (CET 802)	*Sco*	M	Adult	20/05/2016	Dead	Tenerife	2
**I-907/16 (CET 810)**	*Dde*	F	Juvenile	03/07/2016	Alive	Tenerife	2

**I-167/17 (CET 854)**	*Sfr*	F	Juvenile	15/05/2017	Dead	Tenerife	3
**SA038/18 (CET 884)**	*Sfr*	F	Calf	16/01/2018	Dead	Tenerife	2
**SA223/18 (CET 921)**	*Sco*	M	Juvenile	05/07/2018	Dead	Gran Canaria	3
SA256/18 (CET 930)	*Dde*	M	Adult	06/09/2018	Dead	Fuerteventura	2

Table S1. Stranded cetaceans tested for herpesvirus in brain samples. ID: Identification code, Sp: species, sex, AC: age class, SD: stranding date, ST: stranding type, SP: stranding place and CC: carcass condition. Positive animals are indicated in boldface.
